# Web-based guided insulin self-titration in patients with type 2 diabetes: the Di@log study. Design of a cluster randomised controlled trial [TC1316]

**DOI:** 10.1186/1471-2296-10-40

**Published:** 2009-06-09

**Authors:** Mariëlle GA Roek, Laura MC Welschen, Piet J Kostense, Jacqueline M Dekker, Frank J Snoek, Giel Nijpels

**Affiliations:** 1 The EMGO Institute for Health and Care Research, VU University Medical Center, Amsterdam, The Netherlands; 2 Department of General Practice, VU University Medical Center, Amsterdam, The Netherlands; 3 Department of Epidemiology and Biostatistics, VU University Medical Center, Amsterdam, The Netherlands; 4 Department of Medical Psychology, VU University Medical Center, Amsterdam, The Netherlands

## Abstract

**Background:**

Many patients with type 2 diabetes (T2DM) are not able to reach the glycaemic target level of HbA1c < 7.0%, and therefore are at increased risk of developing severe complications. Transition to insulin therapy is one of the obstacles in diabetes management, because of barriers of both patient and health care providers. Patient empowerment, a patient-centred approach, is vital for improving diabetes management. We developed a web-based self-management programme for insulin titration in T2DM patients. The aim of our study is to investigate if this internet programme helps to improve glycaemic control more effectively than usual care.

**Methods/Design:**

T2DM patients (n = 248), aged 35–75 years, with an HbA1c ≥ 7.0%, eligible for treatment with insulin and able to use the internet will be selected from general practices in two different regions in the Netherlands. Cluster randomisation will be performed at the level of general practices. Patients in the intervention group will use a self-developed internet programme to assist them in self-titrating insulin. The control group will receive usual care.

Primary outcome is the difference in change in HbA1c between intervention and control group. Secondary outcome measures are quality of life, treatment satisfaction, diabetes self-efficacy and frequency of hypoglycaemic episodes. Results will be analysed according to the intention-to-treat principle.

**Discussion:**

An internet intervention supporting self-titration of insulin therapy in T2DM patients is an innovative patient-centred intervention. The programme provides guided self-monitoring and evaluation of health and self-care behaviours through tailored feedback on input of glucose values. This is expected to result in a better performance of self-titration of insulin and consequently in the improvement of glycaemic control. The patient will be enabled to 'discover and use his or her own ability to gain mastery over his/her diabetes' and therefore patient empowerment will increase. Based on the self-regulation theory of Leventhal, we hypothesize that additional benefits will be achieved in terms of increases in treatment satisfaction, quality of life and self-efficacy.

**Trial registration:**

Dutch Trial Register TC1316.

## Background

The prevalence and incidence of type 2 diabetes mellitus (T2DM) is high and the number of persons with T2DM is growing rapidly to be 366 million in 2030 [1]. International guidelines recommend tight glycaemic control, in order to prevent the onset or to reduce the progression of complications associated with T2DM [2-5]. However, achieving tight glycaemic targets represents a major challenge. A Dutch study found that at least 30 percent of T2DM patients under care of General Practitioners (GPs) do not achieve good glycaemic control [6].

Insulin therapy should be started when other therapies fail to reach the glycaemic target of HbA1c < 7.0% [5]. Nonetheless, both patients and health care providers often appear reluctant to start insulin therapy [7-9]. GPs largely do not feel familiar with the perceived complexity of the insulin treatment regimen or they think it is too time consuming [7,8]. Patients as well as health professionals fear negative side effects like weight gain and hypoglycaemia feeding into "psychological insulin resistance", causing unwanted delay of insulin initiation [9]. Therefore, it is important to develop tools that facilitate the transition to insulin therapy with subsequent positive effects on glycaemic control. Interactive Behaviour Change Technology (IBCT), including the use of hardware and software to promote and sustain behaviour changes, could provide such a tool [10], and make the titration of insulin become easier for both the patient and the health care provider. Moreover, a patient-driven insulin titration has already proven to be successful [11]. With IBCT in the form of an internet programme, self-titration (i.e. self-monitoring of blood glucose and self-adjustment of insulin dose) could be further facilitated.

### Web-based diabetes management

IBCT is one potential resource for improving diabetes management. In general it assists patients and their clinicians in monitoring changes in health and self-care needs. Secondly, it supports patients' efforts to make behaviour changes by promoting health and effective self-care, and thirdly it enhances communication between patients and potential supports for their disease management. IBCT increases patients' access to the types of services available from their health care team [10].

Several reviews about utilization of IBCT applications to improve care of chronic illness have been published, and these generally have been positive [10]. A systematic review assessing the effects of IBCT for people with a chronic disease found that IBCTs appeared to improve users' knowledge, social support, health behaviours, self-efficacy (a person's belief in their capacity to perform specific skills in a specific situation) and clinical outcomes [12]. However, the included studies involved different IBCTs with different characteristics, for a wide range of chronic diseases.

The use of IBCT in diabetes care has been mainly focussed on the improvement of glycaemic control. Several studies found promising results making use of different aspects of the opportunities of IBCT: improving communication and computerized educational programs [13,14], or making use of a web-based glucose monitoring system [15-19]. A meta-analysis of 16 studies in which home glucose records were used to perform computer-assisted insulin dose adjustment by clinicians showed a significant improvement of HbA1c [20]. To our knowledge, computer-assisted insulin self-titration has not yet been studied in (previous insulin-naive) T2DM patients. In addition, the causal pathways between supposed improved outcomes and IBCT applications in diabetes care remained unclear, because of lack of clarity in how technological innovations of IBCTs were defined and how their impact was measured [21]. In this study we will investigate the use of an IBCT application in T2DM patients based on a theoretical framework for a better understanding and interpretation of the outcomes.

### Theoretical background

The self-titration of insulin supported by an internet programme is based on the patient empowerment approach, defined as 'helping people to discover and use their own ability to gain mastery over their diabetes' [22]. The key element of patient empowerment in diabetes is to facilitate self-management [22,23]. Diabetes outcomes are largely dependent on the daily self-care activities of the patient [24-26]. Empowering patients to better understand and self-manage their diabetes therefore is key, to achieve satisfactory diabetes outcomes, quality of life, satisfaction with treatment and better communication with caregivers [27-29]. The benefits for the caregivers are an increased satisfaction in work, and achievement of treatment goals [29].

Building on the patient empowerment approach, we developed the content and process of our study guided by the principles of the self-regulation theory. This theory was elaborated by Leventhal and colleagues and it proposes that individuals will use strategies that are based on the understanding of their illness and new experiences [30,31]. The theory delineates five core dimensions of illness representations (peoples' perceptions of and beliefs about an illness): identity, cause, timeline, consequences and controllability of the disease in terms of prevention and cure. Through experience and feedback mechanisms, perceptions can be influenced [32]. Web-based support programmes can provide instant and constructive feedback. Illness perceptions could change and confidence and autonomy could increase. Higher self-efficacy beliefs and higher control perceptions are associated with better metabolic control [33].

In our study we will investigate a patient-centred internet intervention supporting self-titration of insulin therapy in T2DM patients. The internet programme will promote self-regulatory behaviour by effective self-monitoring and evaluation of self-care behaviours through feedback on input of the patients' glucose values. This is expected to result in improved self-management skills, self-efficacy and subsequent glycaemic control. Successful self-regulation of diabetes is expected to translate into better quality of life and treatment satisfaction compared to the control group, where there is less emphasis on patients' self-management.

### Objectives

The primary objective of the study is to determine the effect on glycaemic control (HbA1c) of a patient-centred web-based insulin-titration programme in suboptimal controlled T2DM patients starting insulin treatment.

Secondary objectives are to assess the effects of the intervention on frequency of hypoglycaemic episodes, illness perceptions, self-efficacy, treatment satisfaction, and quality of life.

## Methods/Design

### Design of the study

The design of the study will be a cluster randomised controlled trial at the level of general practices in order to eliminate the influence of contamination of treating patients from both the intervention and control group at the same time. The GP or practice nurse can become more conscious of the treatment process and therefore be stimulated to improve their usual care for the control group as well. The Medical Ethics Committee of the VU University Medical Center in Amsterdam approved the study design, protocols, information letters and informed consent form.

### Setting

Participants will be recruited from general practitioners in the region of Amsterdam and Twente in The Netherlands. A pilot study among diabetes nurses (n = 2) and diabetes patients using insulin (n = 4) from the Diabetes Research Center VUmc in Hoorn has preceded the intervention to test the user friendliness and content of the internet programme. The user friendliness was experienced as good by means that both the nurses and patients were satisfied about the content of the program and were able to use all aspects of the programme easily. They also had the opinion that the program could be used by people not very familiar with using the internet. Only some textual changes in the software were made. The present manuscript can be regarded as the definitive study protocol.

### Study population

The target population consists of T2DM patients (35–75 years) from general practitioners with suboptimal controlled glucose (i.e. HbA1c ≥ 7.0%) and maximal oral hypoglycaemic agents, not using insulin. Inclusion and exclusion criteria are listed in Table 1.

**Table 1:** Inclusion and exclusion criteria

**Inclusion criteria**
• Type 2 diabetes mellitus patients from selected general practices • Between 35 and 75 years • HbA1c ≥ 7.0% in combination with maximal oral hypoglycaemic agents (i.e. the combination of two oral medicines, what cannot further be increased) • Used to a computer and used to the internet • Ability and willingness to inject insulin • Ability and willingness to perform self monitoring of blood glucose • Written informed consent • Understanding of Dutch language
**Exclusion criteria**
• Serious cognitive impairment • Serious other endocrine disorders • Serious disease with a life expectancy < 1 year • Corticosteroid use

### Randomisation and treatment allocation

General practices will be randomly assigned to the intervention or control group using a computerized randomisation programme. In case a practice nurse takes care of the insulin titration (working for one or more general practices), the practice nurse will be randomised and corresponding general practice(s) will be allocated to one of the groups. It is desirable that the two groups will be similar with regard to the amount of patients in each group. For that reason we will apply stratified randomisation [34]. Clusters with one or two general practices and clusters with three or more general practices will be randomised separately. Randomisation will be performed by the manager of the website company (Curavista B.V., Geertruidenberg, the Netherlands), who is independent of the patients and their care. Patients in the intervention group will self-adjust the insulin dose supported by an internet programme and receive education before starting insulin. The control group will receive physician-driven (GP or practice nurse) adjustments, i.e. the GP or practice nurse will perform the insulin titration in his/her own manner, guided by guidelines from the Dutch College of General Practitioners.

When a patient is eligible to participate in our study the GP or practice nurse will provide a letter of invitation and an information brochure of the trial based on the assigned group. The next visit will be scheduled one week later in which the patient can give his or her informed consent to participate in the trial.

**Figure 1:**
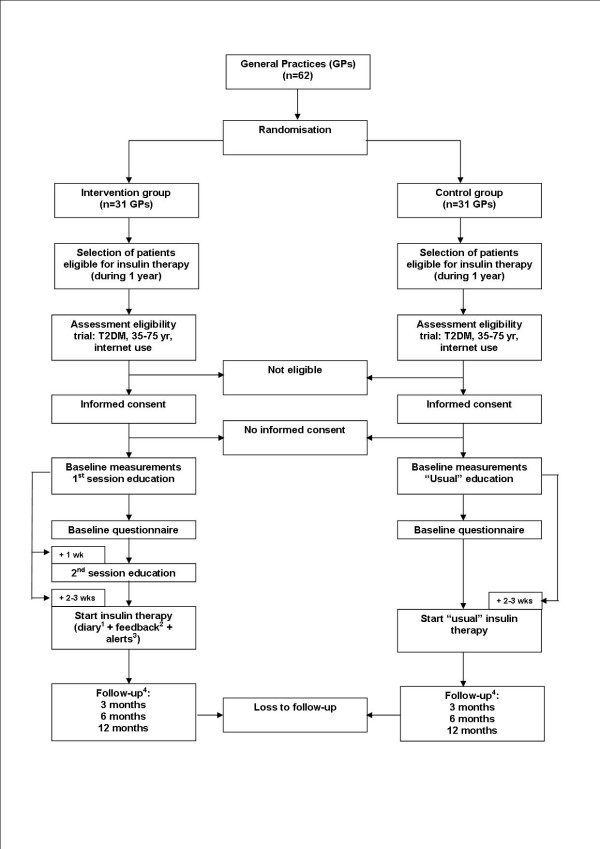
**Flowchart of the study**. ^1 ^The online diary is used for monitoring blood glucose measurements. ^2 ^Feedback consistsof 1. a graphic presentation of the input 2. a dose advice for thenext 2 days or coming period 3. compliments/advice. ^3 ^Alerts are generated when there is a medical urgency: hypoglycaemia (< 2.5 mmol/l) or hyperglycaemia (FBG > 20 mmol/l). When the patient gets an alert (feedback and advice), this is also sent to the GP. ^4 ^Follow up consists of questionnaires and measurements of physical and clinical characteristics (see text: 'outcome assessment').

The flow of the patients is registered by the investigator (MR), according to a flow diagram recommended by the CONSORT statement and its extended version to cluster randomised trials [35,36]. Reasons for withdrawal are registered by the practice nurse or GP. Figure 1 shows the design of the study.

### Blinding

It is impossible to blind the participants and health care providers (GP and practice nurse) for the intervention. The investigators will remain blinded during the entire intervention.

### Study procedure

Approximately 62 GPs (see 'sample size calculation') will be recruited by the principle investigator (MR) and thereafter randomly assigned to the intervention group or control group. Their patients will be allocated to the assigned group.

In the present study, eligible patients will receive insulin glargine. Once-daily injection of a long-acting insulin is attractive, because of the simple dose adjustments based on fasting blood glucose values. It provides at least equivalent glycaemic control to NPH insulin but with a lower incidence of hypoglycaemia [5,25,37]. The GP is free to continue all oral agents, except for thiazolidediones [4].

#### Intervention group

When informed consent is given, patients in the intervention group will receive individual education on diabetes in general, all aspects of insulin treatment, the use of a self-monitoring device, the importance of self-monitoring, and aspects of hypo- and hyperglycaemia. In addition, information about diabetes in general and its management is available on the trial website (provided by [38]), an online education programme developed by the University of Maastricht and the Academic Hospital Maastricht, the Netherlands). The education will be given in two sessions (with an interval of 1 or 2 weeks) by the GP or practice nurse in general practices using a standard protocol provided by the investigators. The patients will receive a manual how to use the internet programme.

The internet programme is accessible through a log-in procedure, requiring a log-in name and a password. The programme is not accessible for the control group or others without permission. In case of problems, patients can always contact their practice nurse or GP, who do have access to the online data of their patients.

Patients will start with 10 IE insulin glargine. The next day, a patient has to log-in in order to start the internet programme, that consists of an online-diary with computerized algorithms. Feedback will be given on fasting blood glucose (FBG) measurements that have to be completed in the diary. The programme will reply with an insulin dose advice when two FBG measurements (on two successive days) are inputted (+ 4 IE when FBG exceeds 10 mmol/l for 2 consecutive days; + 2 IE when FBG is between 7–10 mmol/l for 2 consecutive days; – 2 IE when FBG is between 2.5–4 mmol/l for 2 consecutive days; – 4 IE when FBG is below 2.5 mmol/l for 2 consecutive days). In case of a high or low FBG level, the programme will also respond with a feedback question, guided by the self-regulation theory. According to the patients' answer, advice will be given on the concerning item (e.g. adjustment of diet or increase of physical activity). Feedback will also be given visually in colours, tables and graphics.

The process of using the online-diary will continue until the patient has reached a normal FBG (FBG between 4.0 and 7.0 mmol/L). At that point he/she is advised to make a 5-point day-curve. Dependent on the value of one of the measured glucose values, the internet programme will automatically respond with a feedback question or will give advice to repeat the day-curve after 2 days. When a stable insulin dose is reached (all measurements are within the range of 4.0 – 9.0 mmol/L), it is advised to measure FBG once a week. When repeated day-curves (4 day-curves in approximately one week) are not within normal range, it is advised to contact the GP or practice nurse. Protocols for how to act in different situations will be provided to the GP's. If another or additional (short-acting) insulin is started, patients will not be able to use the internet programme any longer. This is also the case if the insulin dose exceeds 80 IE. If the GP or practice nurse needs extra advice, a diabetes nurse from the Diabetes Research Center can be contacted.

#### Control group

Patients in the control group will receive individual instructions with regard to insulin dose-adjustments from their GP or practice nurse as usual. The number or type of contacts might differ per practice. The titration scheme of insulin glargine will be determined by the GP or practice nurse. Because there is no strict regimen, this can also differ per practice. The patients are offered access to the information provided on the trial website and for completing the web-based questionnaires only. GPs or practice nurses can contact a diabetes nurse from the Diabetes Research Center concerning questions about the insulin therapy.

### Outcome assessment

Outcome measurements are assessed by means of self-administered web-based questionnaires (accessible with a log-in name and password provided by email for both the intervention and the control group) and physical examination at the general practices. If questionnaires are not completed within one week an email-reminder will be sent to the participants. In case of no response, a phone call to their general practice will be made by the investigator. Physical and clinical data will be obtained from the usual 3-monthly check-ups for diabetes patients in their own practice. The practice nurse or GP will record patients' diabetes duration, co-morbidity, pre-existing diabetes complications, and medication at baseline.

#### Primary outcome measure

The primary outcome measure is the difference in change in glycaemic control (HbA1c) between intervention and control group. HbA1c will be measured at baseline, 3 and 12 months. The measurement of HbA1c at 6 or 9 months will take place only if HbA1c is still above 7% at 3 respectively 6 months.

#### Secondary outcome measures

The following secondary outcomes will be assessed in web-based self-administered questionnaires:

• Quality of life is assessed with the 12-item Short Form Health Survey (SF-12) [39], measured at baseline and after 3 and 12 months. The EuroQol (EQ-5D) [40] will be administered at baseline and after 3, 6 and 12 months. This questionnaire assessing the current health status consists of 5 dimensions (mobility, self-care, usual activities, pain/discomfort, and anxiety/depression), each with 3 levels.

• Treatment satisfaction is assessed with the Diabetes Treatment Satisfaction Questionnaire status and change version (DTSQs and DTSQc) [41-44]. The 8-item questionnaires, measure treatment satisfaction and how this satisfaction has changed on 7-point Likert scale. It will be used as an evaluation instrument of the intervention and will be measured at baseline, 3 and 12 months.

• Self-efficacy beliefs are assessed with the Confidence in Diabetes Self Care. This is a 21-item questionnaire, measuring the level of confidence a diabetes patient has in performing self-care activities on 5-point Likert scale [45]. It will be measured at baseline, 3, 6 and 12 months and will be used in evaluating the theoretical background.

• Illness perceptions are assessed with the brief Illness Perception Questionnaire (brief-IPQ). This is a 9-item questionnaire assessing the five core dimensions of illness representations (illness identity, timeline, personal control, treatment control, cause) according to Leventhal's theory of self-regulation [46]. It will be measured at baseline, 3, 6 and 12 months to determine changes in patients' representations regarding diabetes and its controllability and will also be used in evaluating the theoretical background.

• The patient-reported number of hypoglycaemias will be measured by means of a hypoglycaemia diary in which the patient report the severity of each event, glucose value, self-treatment and need of assistance.

#### Other data collection

• *Patient characteristics, internet use (assessed on baseline):* demographic variables (age, gender, marital status, nationality, socio-economic state); experience in use of the internet; smoking (cigarettes/day) and alcohol (glasses/day) use.

• *Medication, diabetes care use:* Total required insulin dose (every 3 months this will be self-reported in the web-based questionnaire); Time delay to reach stable insulin dose (i.e. HbA1c < 7.0%); (Oral) medication changes (data obtained from pharmacy and GP); Frequency of contacts with health care providers (every 3 months this will be self-reported in the web-based questionnaire).

• *Physical and clinical measurements (assessed on the usual diabetes check-ups in the general practices every 3 months):* Weight; Length; Blood pressure; FPG; Lipid spectrum: triglycerides, total cholesterol, and HDL- and LDL-cholesterol (assessed at baseline and after 12 months).

• *Depression (assessed on baseline, 3 and 12 months):* The Patient Health Questionnaire is used to assess the general health of the patient. This brief PHQ (PHQ-9) consists of 9 items, measured on a four-point scale, in order to assess depressive disorders during the last two weeks [47]. This will be assessed because of the possible confounding effect depression has on self-performing activities.

• *Insulin Perceptions (assessed on baseline, 6 and 12 months):* Negative perceptions in insulin naive and insulin-treated patients regarding insulin treatment and changes therein are assessed with the Insulin Initiation Perception Scale (IIPS), a short version of the Insulin Treatment Appraisal Scale (ITAS) [48].

### Sample size calculation

The sample size is calculated to detect a clinical relevant difference of 0.5% in HbA1c between groups. In the Diabetes Care System West-Friesland the standard deviation (sd) of HbA1c of the general diabetes population is 1.2% [49]. A difference (d) between the intervention and control group in changes of 0.5%, a standard deviation (sd) of 1.2%, an alpha of 0.05 (two-sided), and a power of 80%, the number of patients requested in each group is 90.

Because cluster randomisation is applied at the level of general practices, the number of patients has to be multiplied with the following formula: 1 + (k-1)ρ, in which ρ is the intercorrelation coefficient (ICC) between practices. The ICC is statistically determined to be 0.05. k is the number of patients per practice that will join the study. Based on our experience, we estimate that 4 patients per practice will make the transition to insulin treatment in one year and meet the criteria of our trial. A participation of 26 general practices is needed per group:





Furthermore, taking into account a possible dropout rate of 15%, the total number of practices needed is: 2 * 26/(1–0.15) = 62, which means that 248 patients will be required.

### Analysis

Descriptive statistics (means ± SD or median and inter quartile range as appropriate) will be used to describe the study sample with regard to demographics and baseline (clinical) characteristics. On the basis of an intention-to-treat analysis, differences in changes between the intervention group and control group are calculated with 95% confidence intervals at 3, 6 and 12 months for both primary and secondary outcomes.

Using *t*-tests and multiple linear regression – duly adapted for the multilevel structure of the data – we will compare changes in HbA1c, number of hypoglyceamias, quality of life, treatment satisfaction, illness and insulin perceptions and confidence in diabetes self-care scores at different time intervals between the groups, with adjustment for important prognostic factors like age, diabetes duration, medication and level of education where appropriate. Separate analyses of possible effect modifiers (i.e. age, depression, previous internet use) will be performed in order to gain a better understanding as to who benefits most from the intervention.

## Discussion

This article presents a detailed description of a cluster RCT with the aim to investigate the effects of a patient-centred internet programme supporting self-titration of insulin therapy in type 2 diabetes patients compared with standard-of-care physician-driven insulin titration. This will provide researchers and health care providers the opportunity to critically review the methodological quality, the background theory and the practical issues of the RCT [50]. The key element of this trial is that this web-based self-management intervention in the treatment of T2DM patients is designed to enhance patient empowerment, what could result in adequate self-management behaviours (including insulin dose adjustments) that in turn will help to improve and sustain glycaemic control.

Besides the focus on increasing patient empowerment, a strength is the construction of the intervention guided by the self-regulation theory of Leventhal. The use of a theory in general is important for several reasons. A theory helps to design the intervention, it provides a good base for the evaluation of the intervention ('how does it work (or not)?') and it will enable other researchers to refine the theory or intervention [51,52]. In designing the internet programme of our study we have used an important aspect of the self-regulation theory: providing feedback [32]. This will promote effective self-evaluation of health and self-care behaviours and adjustment of cognitive representations and beliefs and subsequent glycaemic control. We will evaluate our theoretical background by investigating if the intervention has increased self-efficacy and changed illness perceptions. Another strength is the use of internet technology. A large part of the population in the Netherlands (86% of all households in 2008) has access to the internet [53]. Internet can meet different needs of patients, like the need of adequate information and continue, access to care [54]. E-health applications are upcoming and the Dutch Patient Consumer Federation (NPCF) has published a vision document, stimulating e-health developments [54].

The intervention will be provided to patients from two different regions in the Netherlands: Amsterdam (an urban region) and Twente (a rural area), which should add to external validity, i.e. generalisability. If our intervention proves to be more effective than care as usual, the internet programme could be widely implemented in general practices in the Netherlands.

There are also some limitations in the study design. We will compare the intervention with usual care. In the Netherlands, most general practitioners or their practice nurses take care of insulin titration, but there is no strict insulin regimen. That means that usual care is not the same in each practice. However, in the sample size calculation we accounted for this by multiplying with an intercorrelation coefficient, providing sufficient power to prevent this bias. Another limitation is the use of different co-medication next to insulin. Except for thiazidediones, metformin and SU-derivates can be continued. That means that there can be some additional effect of oral hypoglycaemic agents on HbA1c. Because we aim at a close approach to the real life situation, the individual GP is free to decide about continuing or stopping the oral medication. Furthermore, the addition of short-acting insulin analogues or even switching to another insulin could be needed in individual patients. At that point the internet programme can not be used for insulin dose advices in the intervention group. All co-medication will be recorded and in analyses used as possible confounder or effect modifier.

The self-titration of insulin and computer-based adjustment of insulin dose has been investigated before. However, the proposed trial is to our knowledge, the first to assess the effects of a web-based guided insulin self-titration intervention in previous insulin-naive T2DM patients. It can make a considerable contribution to the evidence of the importance of the patient empowerment approach in improving diabetes care, making use of modern technology.

The trial has started in January 2009 and the first results will be available in 2011.

## Abbreviations

T2DM: type 2 diabetes mellitus; HbA1c: glycated haemoglobin; GP: general practitioner; ADA: American Diabetes Association; IBCT: interactive behaviour change technology; EASD: European Association for the Study of Diabetes; FPG: fasting plasma glucose.

## Competing interests

JD has received a research grant from Novo Nordisk. FS has received research funds from Sanofi-Aventis.

## Authors' contributions

MR is responsible for the data collection and wrote the manuscript. GN developed the original idea for the study. The study design was further developed by GN, LW and MR. All authors have read and approved the final manuscript.

## Pre-publication history

The pre-publication history for this paper can be accessed here:


